# Performance and Safety of Amino-Acid- and Hydroxyapatite Enriched-Hyaluronic Acid Intradermal Gel in Facial Skin Defects

**DOI:** 10.3390/medicina60071121

**Published:** 2024-07-11

**Authors:** Salvatore Todde, Fabiano Svolacchia, Lorenzo Svolacchia, Federica Giuzio, Sameer Kumar Panda, Giuseppe A. Ferraro

**Affiliations:** 1 Plastic Surgery Unit, Multidisciplinary Department of Medical-Surgical and Dental Specialties, University of Campania Luigi Vanvitelli, 80138 Naples, Italy; saltod89@gmail.com; 2 Departments of Medical-Surgical Sciences and Biotechnologies, Sapienza University of Rome, 00118 Rome, Italy; fabiano.svolacchia@gmail.com (F.S.); lorenzo.svolacchia@hotmail.it (L.S.); 3 Department of Sciences, University of Basilicata, Via Nazario Sauro 85, 85100 Potenza, Italy; federica.giuzio@unibas.it; 4 U.O.S.D. of Plastic Surgery A.O.R “San Carlo”, 85100 Potenza, Italy; 5 Department of Experimental Medicine, University of Campania Luigi Vanvitelli, Via L. Armanni, 80138 Naples, Italy; 6 Dipartimento di Medicina e di Scienze della Salute “Vincenzo Tiberio”, Cattedra di Chirurgia Plastica Ricostruttiva ed Estetica, Università Degli Studi del Molise, 86100 Campobasso, Italy; giuseppe.ferraro@unimol.it

**Keywords:** intradermal gel, hyaluronic acid, hydroxyapatite, facial skin aging, extracellular matrix, Peptidyal 2, Peptidyal HX, Doublyx Evo

## Abstract

*Background and Objectives*: The facial skin defects associated with aging are common concerns in the aging population. Hyaluronic-acid-based intradermal gels have established themselves as safe and effective treatments for addressing these concerns. Recently developed enriched products aim to enhance the efficacy of these gels, yet their effectiveness lacks thorough validation in the existing literature. *Materials and Methods*: In this retrospective analysis, we investigated the outcomes of intradermal gel treatments in 103 patients with soft tissue defects. This study included three groups: 35 patients received amino-acid-enriched hyaluronic acid gel, another 35 were treated with hydroxyapatite-enriched hyaluronic acid gel, and the remaining 33 underwent hyaluronic acid treatment only. The efficacy of the treatments was assessed using the Global Aesthetic Improvement Scale (GAIS) score, while patient satisfaction was gauged through a detailed questionnaire. Any adverse event was monitored. *Results*: The treatments demonstrated remarkable efficacy, as evidenced by mean GAIS scores of 1.714 points for those treated with amino acid-enriched hyaluronic acid gel, 1.886 points for individuals receiving hydroxyapatite-enriched hyaluronic acid gel, and 1.697 for those treated with hyaluronic acid alone, all showing statistical significance (*p* < 0.0001). Patient satisfaction was very high. Significantly, there were no recorded instances of major adverse events. *Conclusions*: Hyaluronic gels, particularly those enriched with amino acids and hydroxyapatite, are effective and safe interventions for addressing facial skin aging defects. They serve as valuable tools in mitigating age-related blemishes and contribute to the overall improvement of skin aesthetics.

## 1. Introduction

Skin aging is intricately influenced by the interplay of extrinsic and intrinsic factors, with environmental aggressors such as solar ultraviolet (UV) radiation and genetically influenced intrinsic changes being prominent contributors to this multifaceted process [1,2]. These mechanisms converge on common molecular and cellular pathways, notably generating reactive oxygen species (ROS) [3,4,5]. Additionally, aging profoundly impacts the extracellular matrix (ECM), impairing its ability to synthesize and catabolize essential components such as collagen, elastin, and glycosaminoglycans (GAGs). Consequently, these processes culminate in a diminished volume and richness of the ECM, rendering it more susceptible to heightened enzymatic degradation by metalloproteinases and collagenases [5,6]. Moreover, during the natural aging process, a decline in endogenous hyaluronic acid (HA) levels leads to diminished skin hydration and, subsequently, reduced skin elasticity. This phenomenon manifests itself as soft tissue deficits, notably influencing the skin’s physiological characteristics and aesthetic appearance [7,8].

Injectable gels have emerged as longstanding interventions to address age-related soft tissue defects stemming from these molecular and cellular alterations. Predominantly formulated with HA, these gels have been employed for decades to correct age-associated soft tissue deficiencies. Currently recognized as the gold standard for such treatments, these HA-based injectable gels have demonstrated a commendable track record of safety, efficacy, and user-friendliness [9,10,11]. The unique combination of biocompatibility, biodegradability, hygroscopicity, viscoelasticity, and reversibility establishes HA as a prominent choice for soft tissue augmentation [12,13,14,15,16,17]. These distinctive properties enhance the safety profile of HA-based interventions and contribute to their efficacy and versatility in achieving enduring aesthetic results.

In addressing skin aging, HA’s properties can be enhanced through supplementation with synergistic elements, such as amino acids and hydroxyapatite. Amino acids are pivotal in supporting collagen synthesis, a crucial aspect of maintaining skin elasticity and firmness [18,19]. Furthermore, amino acids possess moisturizing properties, fostering improved hydration and promoting a balanced skin texture. Hydroxyapatite is a mineral that constitutes a primary component of bones and teeth, finding utility in cosmetic medicine [20,21,22]. Hydroxyapatite’s biocompatibility, osteoconductive properties, durability, controlled biodegradation, and versatility contribute to its utility, especially in correcting facial defects. 

While promising, the efficacy of treatments involving HA supplemented with amino acids or hydroxyapatite must be systematically evaluated in clinical practice to establish their effectiveness in addressing age-related skin defects. This study, therefore, holds significant importance as it aims to fill this gap by evaluating the performance of two medical treatments, one containing HA enriched with amino acids (Peptidyal 2) and the other containing HA enriched with hydroxyapatite (Peptidyal HX), in the treatment of soft tissue deficits. To enhance the impact of the results and ensure comprehensive information, we have included another medical treatment exclusively containing HA (Doublyx Evo) in this analysis.

## 2. Materials and Methods

### 2.1. Study Design

This monocentric, investigator-initiated, retrospective, observational study aimed at assessing the safety and efficacy of three medical treatments—Peptidyal 2, Peptidyal HX, and Doublyx Evo—in treating soft tissue deficits. This study involved a comprehensive analysis of patients treated between December 2022 and April 2023 at the University of Campania Luigi Vanvitelli, Naples, Italy. Ethical approval was obtained from the Ethical Committee of 2022 University of Campania Luigi Vanvitelli, Prot. 0038554/i of 22 December 2022. All procedures adhered to the ethical standards set forth by the responsible committee on human experimentation (both institutional and national) and the Helsinki Declaration of 1975, as revised in 2008.

### 2.2. Study Population

This study comprised 103 adult subjects. Of that total, 35 were treated with Peptidyal 2, 35 with Peptidyal HX, and 33 with Doublyx Evo. Patients with incomplete medical records were excluded. Inclusion criteria were adult patients with soft tissue deficiency treated with one of the medical treatments studied, treatment carried out within a time window of 3–9 months before the start of the study, and a written privacy policy consent. The exclusion criteria were as follows: age over 65; patients with severe obesity; patients with previous aesthetic or surgical treatments at the same anatomical site; or patients with concomitant diseases that may have affected the treatment outcome.

### 2.3. Data Collection

Data were collected retrospectively from medical records, focusing on the treatment regimen for each treatment. Eligible subjects underwent a single injection of one of the investigated treatments in areas such as nasolabial folds, lips, midface, perioral, and periocular regions requiring correction. The investigators estimated the injection volume using commercially available hyaluronidase in cases of overcorrection, which was administered at the investigators’ discretion. Post-injection, subjects were instructed to refrain from applying makeup for 12 h, avoid prolonged exposure to sunlight and UV light for 36 h, and abstain from saunas or Turkish baths for one week. Additionally, subjects were advised against massaging the treatment site or applying pressure to the area for one-week post-injection. For all medical treatments, patients underwent treatment within a 30- to 90-day window before the study began.

The treatment protocol involved using the Global Aesthetic Improvement Scale (GAIS) and a satisfaction questionnaire. GAIS, a 5-point scale, assessed global aesthetic improvement in appearance as perceived by the investigator, categorized as “worse”, “no change”, “improved”, “much improved”, and “very much improved” [23,24]. Subject satisfaction was gauged through a series of questions on 3- or 5-point scales (Table 1).

### 2.4. Clinical Investigation Endpoints

All complications and outcomes were reviewed from patients’ medical records.

Primary efficacy endpoint was evaluating the treatment efficacy with the GAIS (Table 2), with a median GAIS score < 4 considered successful. Secondary endpoint was patient satisfaction was assessed using specific questionnaires. Safety endpoint: For the safety analysis, adverse events (AEs) were coded with the Medical Dictionary for Regulatory Activities (MedDRA) version 16.0 terminology and summarized by system organ class and preferred term. Adverse treatment effects and serious adverse events (SADEs), as well as adverse events leading to withdrawal, were summarized separately.

### 2.5. Medical Treatments

Doublyx Evo (Aerazenlab, Milan, Italy) uses highly purified, not cross-linked, 20 mg/mL sodium hyaluronate derived from bacterial fermentation. Hyaluronic acid is known for its biocompatibility, biodegradability, hygroscopicity, viscoelasticity, and reversibility. It effectively addresses soft tissue deficits by providing volume and hydration to the skin, thus improving skin elasticity and reducing the appearance of wrinkles.

Peptidyal 2 (Aerazenlab, Milan, Italy) is made using highly purified, not cross-linked, 20 mg/mL sodium hyaluronate derived from bacterial fermentation, and L-hydroxyproline, L-proline, and glycine amino acids. Amino acids play a crucial role in supporting collagen synthesis, which is essential for maintaining skin elasticity and firmness. Additionally, they possess moisturizing properties that foster improved hydration and promote a balanced skin texture.

Peptidyal HX (Aerazenlab, Milan, Italy) is made using highly purified, not cross-linked, 18 mg/mL sodium hyaluronate derived from bacterial fermentation and hydroxyapatite (up to 0.01%) with glycine and L-proline amino acids. Hydroxyapatite is a mineral that is a primary component of bones and teeth. Its biocompatibility and durability make it useful in cosmetic medicine, particularly for correcting facial defects. It contributes to enhanced collagen production and provides a scaffold for new tissue formation.

All the hydrogels are transparent, colorless, and low-viscosity. All three treatments are supplied in 2.5 mL pre-filled disposable syringes with a luer-lock. These products bear the European conformity mark and have been available on the European market since 2016. These treatments were used separately in this study. The images of all the three gels with their appearances are provided in Figure S1: Gel Images.

## 3. Results

### 3.1. Data Analysis

The sample size was calculated based on estimates of the possible improvement of the GAIS score after treatment using information from the literature. The sample size calculation was based on a continuous response variable, not assuming a normal distribution, specifically the change in the median GAIS score after treatment. The calculations considered an effect size (dz) of 0.50, aiming for a power of 80%. The analysis of the GAIS score utilized a Wilcoxon signed rank test with a two-sided significance level set at 5%. This approach was chosen due to the non-normal distribution assumption. For all other endpoints, a descriptive analysis was employed. This included the assessment of patient satisfaction questionnaire responses and safety endpoints, where adverse events (AEs) were descriptively evaluated.

### 3.2. Patients

A total of 103 subjects underwent screening and were enrolled in the study, with 35 individuals receiving treatment with Peptidyal 2, 35 with Peptidyal HX, and 33 with Doublyx Evo. Subjects treated with Peptidyal 2 were evaluated between 36 and 78 days (median = 41) after treatment (mean = 49.2; STD = 10.3), subjects treated with Peptidyal HX were evaluated between 32 and 77 days (median = 43) after treatment (mean = 51.1; STD = 14.1), and patients treated with Doublyx Evo were evaluated between 33 and 71 days (median = 39) after treatment (mean = 42.5; STD = 12.7). Demographic details are summarized in Table 3. This study exclusively comprised women participants. For those treated with Peptidyal 2, ages ranged from 32 to 60 years, with a median age of 45 years. Body mass index (BMI) varied from 16.46 to 24.01, with a median of 20.35. Similarly, subjects treated with Peptidyal HX had an age range of 26 to 60 years, with a median age of 46 years, and a BMI range of 16.76 to 21.35, with a median of 18.79. Subjects treated with Doublyx Evo had an age range of 29 to 57 years, with a median age of 43.5 years, and a BMI range of 16.06 to 20.39, with a median of 18.22. All subjects were of Italian and Caucasian descent. None of the participants had a significant medical history. Consistent with standard clinical practice, no antiplatelet agents were taken two days before treatments. Additionally, no rescue medication (hyaluronidase) was administered throughout the study.

### 3.3. Patient Response

The primary performance endpoint, measured by the GAIS score, significantly improved patients’ defects in each treatment group (Table 4). Patients treated with Peptidyal 2 exhibited a median GAIS score of 2, with a mean of 1.714 (*p*-value < 0.0001, STD = 0.51). Similarly, those treated with Peptidyal HX and Doublyx Evo showed median scores of 2, with means of 1.886 (*p*-value < 0.0001, STD = 0.75) and 1.697 (*p*-value < 0.0001, STD = 0.75), respectively. Subject satisfaction was notably high across all groups (Table 5). The satisfaction levels of patients treated with Peptidyal 2, Peptidyal HX, and Doublyx Evo were assessed across multiple parameters. For Peptidyal 2, most subjects reported a significant improvement in appearance, with 37.14% stating it was “very much improved” and 48.57% indicating “much improved”. Furthermore, 88.57% of subjects expressed satisfaction with the treatment, and 97.2% would recommend it. Peptidyal HX recipients similarly experienced positive outcomes, with 31.43% reporting a “very much improved” appearance and 48.57% noting a “much improved” status. A high satisfaction rate of 71.43% was observed, along with a 100% recommendation rate. Doublyx Evo recipients experienced substantial improvements, with 27.27% reporting a “very much improved” appearance and 45.45% indicating “much improved”. Additionally, 60.61% expressed satisfaction, and all subjects (100%) would recommend the treatment. These results attest to a notable level of patient satisfaction across all three treatments, highlighting favorable outcomes and a willingness to recommend them to others.

### 3.4. Safety

The safety analysis provides an overview of adverse events (AEs) categorized by system organ class and preferred term for each investigated medical treatment. For Peptidyal 2, out of 35 subjects, nine events were reported, with injection site hematoma and injection site swelling being the most frequent, constituting 14.2% and 11.4% of the total events, respectively (Table 6). Peptidyal HX exhibited a total of seven events out of 35 subjects, with injection site swelling, and general disorders and administration site conditions being the predominant AEs, representing 8.5% and 25.7% of the total events, respectively (Table 7). In the case of Doublyx Evo, seven events were reported among 33 subjects, with injection site swelling, and general disorders and administration site conditions being the most significant AEs, constituting 12.1% and 21.2% of the total events, respectively (Table 8). Most AEs were of mild intensity and did not require specific treatments, reflecting the overall safety profile of the investigated medical treatments. Notably, no significant adverse events or treatment deficiencies were recorded throughout the study, confirming the safety of Peptidyal 2, Peptidyal HX, and Doublyx Evo in treating soft tissue deficits.

## 4. Discussion

This retrospective study enrolled 103 participants, exclusively women aged 40 to 50, with mild to severe face volume deficiency, presumably influenced by age-related and environmental factors. This study aimed to evaluate the safety and efficacy of three medical treatments, Peptidyal 2, Peptidyal HX, and Doublyx Evo, for addressing soft tissue deficits. This study design allowed for a comprehensive assessment, incorporating demographic details, treatment timelines, and stringent inclusion criteria. A validated assessment instrument, the GAIS, was used in this study. The GAIS was completed independently by the subjects and the physician investigators. A 1 grade or better improvement from the baseline was considered to be a clinically meaningful outcome for this assessment scale.

The primary efficacy endpoint, measured by the GAIS, demonstrated significant improvement in the patients’ defects across all treatment groups. Patients treated with Peptidyal 2, Peptidyal HX, and Doublyx Evo exhibited median GAIS scores of 2, with corresponding means and *p*-values indicating statistically significant improvements. The results were consistent with a notable level of patient satisfaction, as evidenced by high percentages of subjects reporting an improved appearance and expressing satisfaction with the treatment. Recommendations for the treatments were very positive, underscoring the perceived efficacy and patient endorsement of Peptidyal 2, Peptidyal HX, and Doublyx Evo.

The observed improvements in the GAIS scores of the three medical treatments align with those in previous studies evaluating HA-based gels [25,26,27]. Including amino acids in Peptidyal 2 and hydroxyapatite in Peptidyal HX formulations may enhance collagen synthesis and fibroblast stimulation, potentially amplifying the volumizing effects and overall aesthetic improvements. Doublyx Evo, although exclusively containing HA, exhibits comparable efficacy, emphasizing the fundamental role of HA in achieving desired outcomes. These formulations synergistically address soft tissue deficits by leveraging distinct mechanisms, showcasing versatility in achieving aesthetic goals.

The safety analysis revealed adverse events categorized by system organ class and preferred term for each treatment. Adverse effects included injection site swelling, which was an anticipated short-term ADE described in previous studies with related products and is a common ADE for intradermal gels [25,26,28]. The most common ADE in this investigation was injection site hematoma. However, the injection of any intradermal gel can be associated with hematoma formation, amongst other injection site reactions such as burning, itching, or pain (secondary to stretching of cutaneous nerves), erythema, edema, or bruising, even with excellent injection technique [25,29,30]. Notably, most adverse events observed were of mild intensity, aligning with an overall favorable safety profile. Notably, no significant adverse events or treatment deficiencies were recorded, confirming the safety of Peptidyal 2, Peptidyal HX, and Doublyx Evo in treating soft tissue deficits within the studied timeframe.

As with any retrospective study, the inherent limitations include the relatively short follow-up period and potential biases. Notwithstanding, while this study did not have an extensive duration, the provided timeline (evaluations between 36 and 78 days) offers insights into the initial durability of the treatments. This short- to medium-term follow-up duration provides insights into immediate and intermediate outcomes but needs a long-term perspective. Moreover, the absence of a placebo group limits contextualization and causation attribution. These limitations emphasize the need for cautious interpretation and consideration in designing future research in soft tissue augmentation.

## 5. Conclusions

In conclusion, this retrospective study provides valuable insights into the safety and efficacy of Peptidyal 2, Peptidyal HX, and Doublyx Evo in addressing soft tissue deficits. The observed improvements in GAIS scores, high patient satisfaction, and favorable safety profiles suggest that these treatments hold promise in aesthetic medicine. Further research with extended follow-up periods and larger cohorts can offer a more comprehensive understanding of these interventions’ durability and their long-term effects. The favorable results highlighted in this study contribute to advancing the field of soft tissue augmentation, positioning Peptidyal 2, Peptidyal HX, and Doublyx Evo as viable options for practitioners and patients seeking safe and effective aesthetic treatments. This study has limitations, and the data highlight the need for further research with larger sample sizes, longer follow-up periods, and additional quantitative measures.

## Figures and Tables

**Table 1:** Subject satisfaction questionnaire.

Question						
How would you judge the change in your appearance after treatment?	1 Very much improved	2 Much Improved	3 Improved	4 Slightly improved	5 No Change	6 Worsened
How satisfied are you with the treatment?	1 Very satisfied	2 Satisfied	3 Not satisfied			
Would you recommend the treatment to your friends and acquaintances?	1 Yes	2 Perhaps	3 No			

**Table 2:** GAIS score.

Rating		Description
1	Very much improved	Optimal Cosmetic result in this subject
2	Much improved	Marked improvement in appearance from the initial condition, but not completely optimal for this subject
3	Improved	Obvious improvement in appearance from the initial condition, but a re-treatment is indicated
4	No Change	The appearance is essentially the same as the original condition
5	Worse	The appearance is worse than the original condition

**Table 3:** Patients’ demographic data.

Peptidyal 2 (N = 35)		
**Age (years)**	Median	45.0
	Range	32–60
**BMI**	Mean	20.17
	STD	1.7
**Peptidyal HX (N = 35)**		
**Age (years)**	Median	46.0
	Range	26–60
**BMI**	Mean	18.74
	STD	1.1
**Doublyx Evo (N = 33)**		
**Age (years)**	Median	43.5
	Range	29–57
**BMI**	Mean	18.22
	STD	1.6

N = number of subjects, STD = standard deviation, BMI = body mass index.

**Table 4:** Global Aesthetic Improvement Score.

Treatment	Median GAIS Score	Mean GAIS Score	*p* Value
Peptidyal 2	2	1.714	<0.0001
Peptidyal HX	2	1.886	<0.0001
Doublyx Evo	2	1.697	<0.0001

**Table 5:** Patient satisfaction.

Subject Satisfaction Level with Peptidyal 2	
**Satisfaction**	**Number (%) of Subjects**
**Q1—Appearance after treatment (N = 35)**	
very much improved	12 (37.14)
much improved	17 (48.57)
improved	4 (11.43)
slightly improved	2 (2.86)
no change	-
worsened	-
**Q2—Satisfaction with treatment (N = 35)**	
very satisfied	31 (88.57)
satisfied	4 (11.43)
not satisfied	-
**Q3—Recommendation of treatment (N = 35)**	
yes	34 (97.2)
perhaps	1 (2.8)
**Subject Satisfaction Level with Peptidyal HX**	
**Satisfaction**	**Number (%) of Subjects**
**Q1—Appearance after treatment (N = 35)**	
very much improved	11 (31.43)
much improved	17 (48.57)
improved	7 (20.00)
slightly improved	-
no change	-
worsened	-
**Q2—Satisfaction with treatment (N = 35)**	
very satisfied	23 (71.43)
satisfied	12 (28.57)
not satisfied	-
**Q3—Recommendation of treatment (N = 35)**	
yes	35 (100.00)
perhaps	-
**Subject Satisfaction Level with Doublyx Evo**	
**Satisfaction**	**Number (%) of Subjects**
**Q1—Appearance after treatment (N = 35)**	
very much improved	9 (27.27)
much improved	15 (45.45)
improved	7 (21.21)
slightly improved	2 (6.06)
no change	-
worsened	-
**Q2—Satisfaction with treatment (N = 35)**	
very satisfied	20 (60.61)
satisfied	13 (30.39)
not satisfied	-
**Q3—Recommendation of treatment (N = 35)**	
yes	33 (100.00)
perhaps	-

**Table 6:** Adverse events by system organ class and preferred term with Peptidyal 2.

Peptidyal 2			
		All, N = 35	
	n	N’	(% of N)
**Total**	**9**	**8**	**(22.8)**
**General dis. and ad. site cond.**	**9**	**8**	**(22.8)**
Injection site hematoma	5	5	(14.2)
Injection site swelling	4	4	(11.4)

n = number of events, N = number of subjects in the data set, N’ = number of subjects with events.

**Table 7:** Adverse events by system organ class and preferred term with Peptidyal HX.

Peptidyal HX			
		All, N = 35	
	n	N’	(% of N)
**Total**	**7**	**6**	**(17.1)**
**General dis. and ad. site cond.**	**7**	**6**	**(25.7)**
Injection site hematoma	2	2	(5.7)
Injection site swelling	3	3	(8.5)
Injection site pain	1	1	(2.8)
Injection site burning	1	1	(2.8)

n = number of events, N = number of subjects in the data set, N’ = number of subjects with events.

**Table 8:** Adverse events by system organ class and preferred term with Doublyx Evo.

Doublyx Evo			
		All, N = 33	
	n	N’	(% of N)
**Total**	**7**	**6**	**(21.2)**
**General dis. and ad. site cond.**	**7**	**6**	**(21.2)**
Injection site hematoma	3	2	(9.1)
Injection site swelling	4	4	(12.1)

n = number of events, N = number of subjects in the data set, N’ = number of subjects with events.

## Data Availability

The data sets used and/or analyzed during the current study are available from the corresponding author upon reasonable request.

## Supplementary Materials

The following supporting information can be downloaded at: https://www.mdpi.com/article/10.3390/medicina60071121/s1, Figure S1: Gel Images
