# Supplementary figures and images for: Performance and Safety of Amino-Acid- and Hydroxyapatite Enriched-Hyaluronic Acid Intradermal Gel in Facial Skin Defects
Source: Medicina (Kaunas). 2024 Jul 11;60(7):1121. doi: 10.3390/medicina60071121 (PMC11279309; doi:10.3390/medicina60071121)

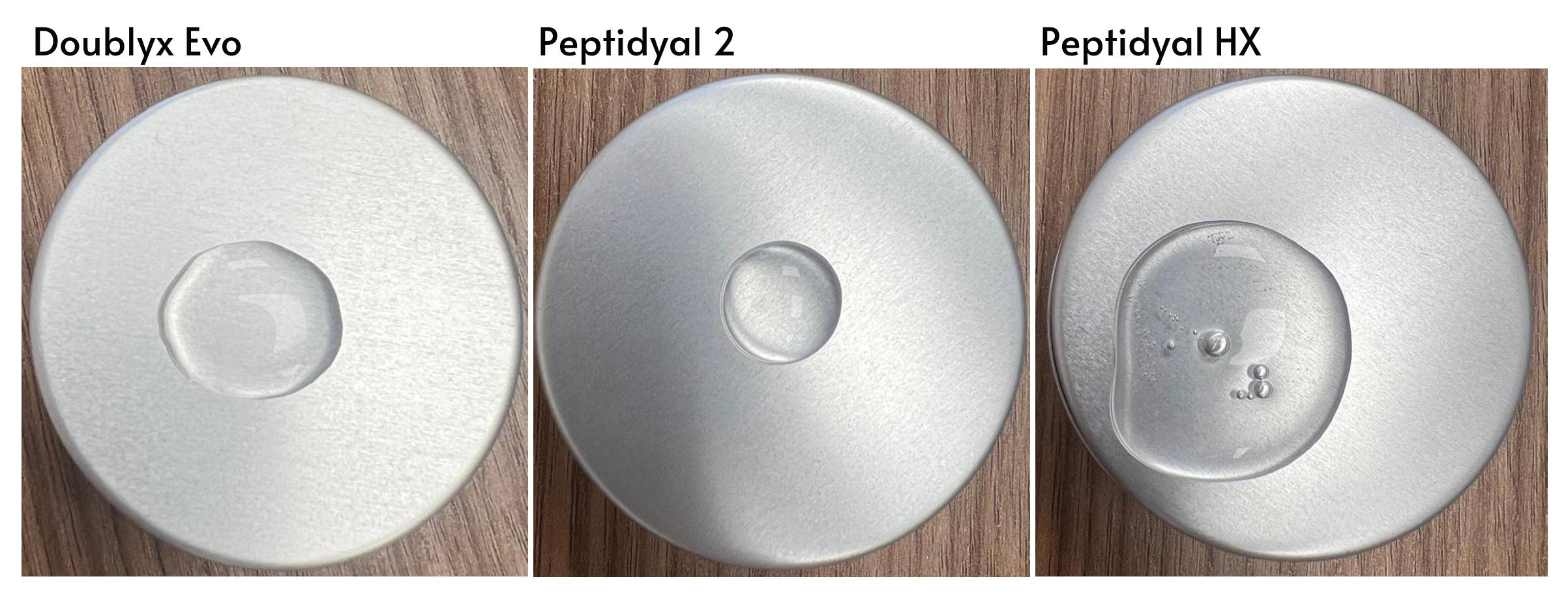

Supplement: Supplementary file 1 [file medicina-60-01121-s001.zip › medicina-3006946-supplementary.tiff]
